# Legionellosis from *Legionella pneumophila* Serogroup 13

**DOI:** 10.3201/eid1109.050345

**Published:** 2005-09

**Authors:** Barzo Faris, Camelia Faris, Mona Schousboe, Christopher H. Heath

**Affiliations:** * North Glasgow University NHS Trust, Glasgow, Scotland, United Kingdom;; † Canterbury Health Laboratories, Christchurch, New Zealand;; ‡ Royal Perth Hospital, Perth, Western Australia, Australia;; § University of Western Australia, Perth, Western Australia, Australia

**Keywords:** Legionella pneumophila serogroup 13, Legionnaires’ disease, non-Legionella pneumophila serogroup 1, near drowning, whirlpool spas, research

## Abstract

We describe 4 cases of *Legionella pneumophila* serogroup 13–associated pneumonia. These cases originate from a broad geographic range that includes Scotland, Australia, and New Zealand. *L. pneumophila* serogroup 13 pneumonia has a clinically diverse spectrum that ranges from relatively mild, community-acquired pneumonia to potentially fatal severe pneumonia with multisystem organ failure. All cases were confirmed by culture and direct fluorescent antibody staining or indirect immunofluorescent antibody tests. Proven or putative sources of *L. pneumophila* serogroup 13 infections in 2 patients included a contaminated whirlpool spa filter and river water. An environmental source was not found in the remaining 2 cases; environmental cultures yielded only other *L. pneumophila* serogroups or nonpneumophila *Legionella* species. We describe the clinical and laboratory features of *L. pneumophila* serogroup 13 infections. *L. pneumophila* serogroup 13 pneumonia is rarely reported, but it may be an underrecognized pathogenic serogroup of *L. pneumophila*.

## Abstract 2

*Legionella pneumophila* serogroup 13 may be underrecognized.

*Legionella* species are relatively common causes of pneumonia (*1*). *Legionella pneumophila* is the most common pathogenic species, with 15 serogroups described. *L. pneumophila* serogroup 1 accounts for most culture-confirmed cases of legionellosis; non–serogroup 1 *L. pneumophila* causes only ≈7% of legionellosis cases (*2*). Legionellae are widely distributed in the environment, including in lakes, rivers, creeks (*1**,**3*), and artificial aquatic habitats such as potable-water supplies (*1**,**3*); amebae are the natural hosts in the environment (*4*).

*L. pneumophila* serogroup 13 was first described as a new pathogenic serogroup of *L. pneumophila* in 1987 (*5*), but a detailed clinical account of these cases was not included in the initial report. *L. pneumophila* serogroup 13 is rarely reported in humans; it accounted for 2 (0.4%) of 508 isolates from a recent international survey of culture-confirmed legionellosis (*2*). Furthermore, a recent European study of 1,335 unrelated clinical isolates yielded only 2 isolates of *L. pneumophila* serogroup 13 (*6*). We are unaware of published reports of *L. pneumophila* serogroup 13 infections in which the epidemiology and the clinical spectrum are outlined. We therefore report 4 cases of *L. pneumophila* serogroup 13 infections to describe in detail these aspects of this uncommon human pathogen.

## The Cases

### Case 1

A 27-year-old woman was admitted to an intensive care unit in Glasgow, Scotland, after she had nearly drowned in estuarine water. On admission, her vital signs were as follows: core body temperature 32.8°C, pulse 92 beats per min, blood pressure 90/65 mm Hg, respiratory rate 28 breaths per min, and Glasgow Coma Scale score 13/15. Arterial blood gas analysis on 100% oxygen showed partial arterial oxygen pressure (PaO_2_) 46 mm Hg (reference range [RR] >90 mm Hg), PaCO_2_ 48 mm Hg (RR 25–35 mm Hg), and bicarbonate 16.2 mmol/L (RR 24–30 mmol/L). Her blood biochemistry showed hyponatremia and hypokalemia. Chest radiograph showed bilateral consolidation. She was intubated, given ventilatory assistance, and actively rewarmed; inotropes were administered, and empiric intravenous cefotaxime and metronidazole were given. With the patient's further clinical deterioration, intravenous vancomycin replaced metronidazole on day 6.

On day 7, high-dose methylprednisolone was administered for worsening respiratory function and chest radiographic evidence of acute respiratory distress syndrome. Given the patient's poor clinical response, on day 14, intravenous clarithromycin and gentamicin were given, with intravenous ciprofloxacin added 24 hours later. Acute renal failure required hemofiltration, and respiratory function deteriorated further on day 15, despite prone ventilation and nitric oxide therapy. On day 18, she died of refractory hypoxemia and multisystem organ failure. A tracheal aspirate collected on day 14 plus postmortem lung tissue samples subsequently yielded colonies of a *Legionella* species on buffered-charcoal yeast extract (BCYE) agar (Oxoid Ltd., Basingstoke, England). *L. pneumophila* serogroup 13 infection was diagnosed by *mip* gene sequencing of the isolate, which demonstrated 99% homology with the type strain of *L. pneumophila* serogroup 13 (GenBank accession no. AF022327). Seroconversion was demonstrated retrospectively by indirect immunofluorescent antibody (IFA) against monoclonal *L. pneumophila* serogroup 13 antisera, with a titer rise from <1:32 on admission to 1:512 on day 12. Domestic or nosocomial sources of legionellosis were not sought. Nevertheless, several months later, estuarine water samples were taken near the site of immersion; cultures were negative for *Legionella* spp.

### Case 2

A 51-year-old man who was undergoing induction chemotherapy for acute myeloid leukemia at Royal Perth Hospital, Australia, was initially given regular and extended periods of home leave in the first week after chemotherapy. However, on day 12 neutropenic typhlitis developed, and a right hemicolectomy was performed. This procedure was followed the next day by the onset of dyspnea, hypoxemia, nonproductive cough, and persistent fever. Chest radiograph showed left lower lobe consolidation, and meropenem with teicoplanin was administered empirically. He initially remained profoundly neutropenic (neutrophil count <0.1 × 10^9^/L [RR 2.0–7.5 × 10^9^/L]). Two days later his hypoxemia and chest radiograph results had worsened, and trimethoprim-sulfamethoxazole, amphotericin B, and roxithromycin were administered. On day 18, he had extensive consolidation that involved most of both lung fields, and his neutrophil count had increased to 2.46 × 10^9^/L.

Bronchoscopic samples collected on day 19 were positive for *Legionella* species antigen by direct immunofluorescent monoclonal antibody stain (DFA) (Genetic Systems, Seattle, WA, USA), but results of his *Legionella* urinary antigen test (Binax Now, Binax, Portland, ME, USA) were negative. Four days later, *Legionella* species were isolated on BCYE agar (Oxoid Ltd.) and confirmed by DFA (Legionella Poly-ID Test Kit, Remel, Lenexa, KS, USA) and also by *L. pneumophila* serogroup 2-14 (LP-2-14) antisera (MarDx Diagnostics, Scotch Plains, NJ, USA). Environmental samples were taken of both hospital and home water. All isolates were then referred to the Australian Legionella Reference Laboratory, Institute of Medical and Veterinary Science, Adelaide, Australia, for typing. Typing confirmed that the clinical isolate was *L. pneumophila* serogroup 13, both by monoclonal antisera (MarDx Diagnostics) and *mip* gene sequencing. Hospital water samples yielded *L. pneumophila* serogroup 10 from both a hand basin cold-water outlet in the patient's room and a cold-water drinking fountain on an adjacent ward. Restriction fragment length polymorphism (RFLP) and pulsed-field gel electrophoresis (PFGE) typing confirmed that the clinical and environmental isolates were genotypically distinct. The patient was then given intravenous erythromycin followed by oral ciprofloxacin for 3 weeks; subsequently, he made a slow but complete recovery. Testing the patient's home potable water supply yielded only *L. feeleii*.

### Case 3

A 48-year-old, previously healthy man was admitted to the hospital in Christchurch, New Zealand, with a 6-day history of increasing dyspnea and a 4-day history of watery diarrhea. He also complained of a dry cough, myalgia, loss of appetite, and poor fluid intake. He appeared flushed and unwell and had dry mucous membranes. His vital signs were the following: temperature 39°C, pulse rate 103 beats per min, blood pressure 154/83 mm Hg, respiratory rate 22 breaths per min, and oxygen saturation 94% on room air. His leukocyte count was 6.1 × 10^9^/L (RR 4.0–10 × 10^9^/L), and his serum sodium and potassium levels were 130 mmol/L (RR 136–146 mmol/L) and 3.3 mmol/L (RR 3.5–5.0 mmol/L), respectively. Liver biochemistry was abnormal. His C-reactive protein (CRP) was >220 mg/L (RR<10 mg/L). Chest radiographs showed pneumonia with segmental consolidation of the lingula and left lower lobe.

The patient's temperature continued to increase to 39.4°C and returned to normal only after 8 days, despite prompt initiation of intravenous amoxicillin and clarithromycin. Liver biochemistry initially deteriorated, peaking on day 6, thereafter slowly returning to normal when the patient was discharged 11 days after admission.

After 6 days of culture on BCYE agar (Oxoid Ltd.), *Legionella* spp. were isolated from sputum collected on the day after admission. The isolate was typed by Environmental Science and Research Limited (ESR), Communicable Diseases Group Laboratory Services, Porirua, Wellington, New Zealand. A strong positive reaction was seen to polyvalent antisera for *L. pneumophila* serogroups 1–14 (Monoclonal Technologies Inc., Alpharetta, GA, USA). Monoclonal antisera (Monoclonal Technologies Inc.) gave a strong positive reaction to *Legionella* strain 82A3105 (CDC 1425-CA-H; ATCC 43736), which identified the isolate as *L. pneumophila* serogroup 13 (D. Harte, pers. comm.).

Public health service investigation of the patient's home yielded *L. pneumophila* serogroup 13 from the filter of an outdoor whirlpool spa. The patient recalled cleaning this filter several days before falling ill. One month later the patient had made a good recovery, although he was easily fatigued.

### Case 4

A 56-year-old man was admitted to the hospital in Christchurch, New Zealand, with a 1-week illness characterized by myalgia, nausea, sweats, and chills but no respiratory symptoms. He was a nonsmoker with negligible alcohol intake; however, his past history included aortic dissection requiring aortic valve replacement.

On admission he was afebrile, blood pressure was 112/66 mm Hg, and his respiratory rate was 16 breaths per min. His chest was clear to auscultation, but his oxygen saturation was 91% on room air, and chest radiograph showed patchy bibasal consolidation. Leukocyte count and serum sodium level were both normal, but his CRP was elevated to 176 mg/L (RR<10 mg/L). Liver biochemistry was slightly abnormal.

He was treated empirically for possible prosthetic valve endocarditis with intravenous penicillin and gentamicin. On the day after admission, he had productive cough and sharp, left-sided chest pains; a temperature of 39°C ensued. Examination showed left basal dullness with bibasal crackles and left basal bronchial breathing. However, he improved rapidly without specific antimicrobial therapy for legionellosis and was discharged 5 days later.

A sputum sample collected on day 2 yielded *Legionella* species after 9 days of incubation on BYCE agar. With the abovementioned methods, ESR typed the isolate as *L. pneumophila* serogroup 13. Extensive investigations by the local public health services yielded *L. micdadei* from spa whirlpool water where the patient regularly swam.

## Discussion

The patients we report had typical signs and symptoms of *Legionella* pneumonia, including headache, anorexia, dry cough, and fever, often with hyponatremia and abnormal results of liver function tests (*7*). Patients had hypoxemia, and disease often involved multiple lobes or both lungs on chest radiograph (*7*). However, these patients generally had minimal preexisting illnesses, apart from the patient with acute myeloid leukemia who was undergoing chemotherapy. The outcome for 3 of our patients was positive, including the patient with an underlying hematologic malignancy and neutropenia; legionellosis is often associated with a markedly elevated death rate in these cases (*3**,**7**,**8*). The patient who died was severely ill after nearly drowning and had aspiration pneumonitis. Moreover, specific therapy that was effective against legionellosis was not started until 14 days after the putative infection; delay in administering appropriate therapy is known to adversely affect outcome (*7**,**9*).

*L. pneumophila* is responsible for ≈90% of infections caused by members of the family Legionellaceae (*1**,**3**,**7*). *L. pneumophila* serogroups 1, 3, 4, and 6 cause most human infections (*1**,**3**,**7*). An international survey found that of 15 serogroups of *L. pneumophila*, 79% of all culture- or urine antigen–confirmed infections were caused by *L. pneumophila* serogroup 1 (*8*). Legionellae are fastidious organisms that are not readily recovered from routine diagnostic media; indeed, an American College of Pathologists' survey indicates that 32% of clinical microbiology laboratories could not grow a pure culture of *L. pneumophila* (*10*). All of our isolates grew only on specialized media. Failure to diagnose legionellosis in many hospital microbiology laboratories is generally due to a limited availability of specialized media and expertise in the culture of legionellae.

In the United States, from 1980–1998, clinicians have increasingly relied on urinary antigen tests to diagnose legionellosis. Use of these methods has led to an increase in *L. pneumophila* serogroup 1 diagnoses from 0% to 69%, with a corresponding decreased frequency of serogroups other than 1 from 38% to 4% (*11*). Urinary antigen tests do not reliably diagnose non–serogroup 1 *L. pneumophila* infections (*12**,**13*), as illustrated by Benson et al., who found sensitivities of 35% (Binax EIA) and 46% (Biotest-EIA), respectively. However, no isolates of *L. pneumophila* serogroup 13 were included in their evaluation (*12*), although as illustrated by 2 of our cases, urinary antigen assays will not likely diagnose this specific serogroup in most cases. Furthermore, in the United States, surveillance systems have found that diagnosis of legionellosis by culture, DFA, and serologic testing (IFA) decreased significantly from 1980 to 1998 (11). Nevertheless, in case 2, the DFA of bronchoalveolar lavage fluid was positive to polyvalent antisera. Also in case 1, IFA serology showed seroconversion to *L. pneumophila* serogroup 13–specific antigen. However, serologic diagnosis of *Legionella* infection has only been fully validated for *L. pneumophila* serogroup 1 (*14*). Thus, the increasing reliance on nonculture-based tests as the sole methods of diagnosing legionellosis is a cause for concern, given the variable utility of these assays.

In most cases, *L. pneumophila* pneumonia is attributed to inhaling contaminated aerosols produced by cooling towers, showers, and nebulizers (*1**,**3**,**7*). Aspiration is also a possible mechanism of transmission (*15**,**16*). The source and reservoir of *L. pneumophila* are generally not identified in sporadic legionellosis (*17**,**18*). However, in case 3, the infection was linked epidemiologically and microbiologically with a contaminated spa whirlpool filter. Although legionellosis is well described in association with spa whirlpools (*19**,**20*), we are unaware of any previously reported cases of *L. pneumophila* serogroup 13 infections linked to spa whirlpools. Additionally, aspirating water and nearly drowning has a rare but well-recognized association with *L. pneumophila* pneumonia (*21**–**23*), although we are unaware of any previously reported cases of *L. pneumophila* serogroup 13 pneumonia after a person's nearly drowning. For the remaining 2 cases, despite extensive environmental sampling, a source or reservoir of infection was not established. In case 2, whether the infection was nosocomial is unclear, given that the patient had had extended periods of home leave. Despite isolation of *L. micdadei* from a spa whirlpool where patient 4 regularly swam, investigations failed to find *L. pneumophila* serogroup 13. Serologic diagnosis of *L. pneumophila* serogroup 13 pneumonia has been occasionally reported from New Zealand and Australia, although clinical data from these cases are unreported. Recovery of environmental isolates mainly from soil and water is also reported in this region (*24**–**26*).

Serotyping, serogrouping, typing, and subtyping legionellae are technically challenging. Both phenotypic and genotypic analyses of *L. pneumophila* are required to reliably epidemiologically link patient and environmental isolates. Phenotypic or genotypic studies in higher reference laboratories were performed on our isolates, thereby reliably confirming their identity as *L. pneumophila* serogroup 13. Epidemiologic studies to identify possible sources of legionellosis require careful investigation, including sensitive and discriminatory subtyping techniques to identify similarities and differences between possibly related strains. Methods reported include various panels of monoclonal antibodies, plasmid analysis, RFLPs, ribotyping, macrorestriction enzyme digestion followed by PFGE, and *mip* gene sequencing (*27*). Recently, Fry et al. have suggested that amplified fragment length polymorphism typing may be the best method for investigating the epidemiology of travel-related legionellosis (*28*). Newer typing techniques for legionellosis include multilocus sequencing typing and DNA chip technologies (*29*).

In summary, we describe for the first time in detail the clinical and laboratory features of *L. pneumophila* serogroup 13 infections. *L. pneumophila* serogroup 13 is a rare but perhaps underrecognized pathogenic *L. pneumophila* serogroup. Although the organism was first reported in the United States (*5*), its global distribution is highlighted here. We found that this organism produces a broad spectrum of clinical disease, from relatively mild disease to severe, potentially fatal pneumonia. Finally, this report emphasizes that culture for *Legionella* species remains important if the prevalence and incidence of legionellosis are to be reliably and fully appreciated.

## References

[1] Fields BS, Benson RF, Besser RE. *Legionella* and Legionnaires' disease: 25 years of investigation. Clin Microbiol Rev. 2002;15:506–26. 10.1128/CMR.15.3.506-526.2002 12097254 PMC118082

[2] Yu VL, Plouffe JF, Castellani-Pastoris M, Stout JE, Schousboe M, Widmer A, Distribution of *Legionella* species and serogroups isolated by culture in patients with sporadic community-acquired legionellosis: an international collaborative survey. J Infect Dis. 2002;186:127–8. 10.1086/341087 12089674

[3] Stout JE, Rihs JD, Yu VL. *Legionella*. In: Murray PR, Baron EJ, Jorgensen JH, Pfaller MA, Yolken RH, editors. Manual of clinical microbiology. 8th ed. Washington: ASM Press; 2003. p. 809–23.

[4] Adeleke A, Pruckler J, Benson R, Rowbotham T, Halablab M, Fields B. *Legionella*-like amebal pathogens—phylogenetic status and possible role in respiratory disease. Emerg Infect Dis. 1996;2:225–30. 10.3201/eid0203.960311 8903235 PMC2626797

[5] Lindquist DS, Nygaard G, Thacker WL, Benson RF, Brenner DJ, Wilkinson HW. Thirteenth serogroup of *Legionella pneumophila* isolated from patients with pneumonia. J Clin Microbiol. 1988;26:586–7. 3356793 10.1128/jcm.26.3.586-587.1988 PMC266340

[6] Helbig JH, Bernander S, Castellani-Pastoris M, Etienne J, Gaia V, Lauwers S, Pan-European study on culture-proven Legionnaires' disease: distribution of *Legionella pneumophila* serogroups and monoclonal subgroups. Eur J Clin Microbiol Infect Dis. 2002;21:710–6. 10.1007/s10096-002-0820-3 12415469

[7] Edelstein PH, Cianciotto NP. *Legionella*. In: Mandell GL, Bennett JE, Dolin R, editors. Principles and practice of infectious diseases. 6th ed. Philadelphia: Churchill Livingstone; 2004. p. 2711–24.

[8] Marston BJ, Lipman HB, Breiman RF. Surveillance for Legionnaires' disease: risk factors for morbidity and mortality. Arch Intern Med. 1994;154:2417–22. 10.1001/archinte.1994.00420210049006 7979837

[9] Heath CH, Grove DI, Looke DF. Delay in appropriate therapy for *Legionella* pneumonia is associated with increased mortality. Eur J Clin Microbiol Infect Dis. 1996;15:286–90. 10.1007/BF01695659 8781878

[10] Edelstein PH. Legionnaires' disease. Clin Infect Dis. 1993;16:741–9. 10.1093/clind/16.6.741 8329504

[11] Benin AL, Benson RF, Besser RE. Trends in Legionnaires' disease, 1980–1998: declining mortality and new patterns of diagnosis. Clin Infect Dis. 2002;35:1039–46. 10.1086/342903 12384836

[12] Benson RF, Tang WT, Fields BS. Evaluation of the Binax and Biotest urinary antigen kits for the detection of Legionnaires' disease due to multiple serogroups and species of *Legionella.* J Clin Microbiol. 2000;38:2763–5. 10878082 10.1128/jcm.38.7.2763-2765.2000 PMC87024

[13] Horn J. Comparison of non-serogroup 1 detection by Biotest and Binax *Legionella* urinary antigen enzyme immunoassays. In: Marre R, Abu Kwaik Y, Bartlett C, Cianciotto NP, Fields BS, Frosch M, et al., editors. *Legionella*. Washington: ASM Press; 2002. p. 207–10.

[14] Wilkinson HW, Cruce DD, Broome CV. Validation of *Legionella pneumophila* indirect immunofluorescent antibody with epidemic sera. J Clin Microbiol. 1981;13:139–46. 7007417 10.1128/jcm.13.1.139-146.1981 PMC273738

[15] Muder RR, Yu VL, Woo A. Mode of transmission of *Legionella pneumophila*: a critical review. Arch Intern Med. 1986;146:1607–12. 10.1001/archinte.1986.00360200183030 3524495

[16] Yu VL. Could aspiration be the major mode of transmission for *Legionella?* Am J Med. 1993;95:13–5. 10.1016/0002-9343(93)90226-F 8328492

[17] England AC III, Fraser DW, Plikaytes BD, Tsai TF, Storch G, Broome CV. Sporadic legionellosis in the United States: the first thousand cases. Ann Intern Med. 1981;94:164–70. 7469207 10.7326/0003-4819-94-2-164

[18] Fiore AE, Nuorti JP, Levine OS, Marx A, Weltman AC, Yeager S, Epidemic Legionnaires' disease two decades later: old sources, new diagnostic methods. Clin Infect Dis. 1998;26:426–33. 10.1086/516309 9502466

[19] Centers for Disease Control and Prevention. Surveillance data from public spa inspections—United States, May–September 2002. MMWR Morb Mortal Wkly Rep. 2004;53:553–5. 15229412

[20] Den Boer JW, Yzerman EPF, Schellekens J, Lettinga KD, Boshuizen HC, Van Steenbergen JE, A large outbreak of Legionnaires' at a flower show, the Netherlands, 1999. Emerg Infect Dis. 2002;8:37–43. 10.3201/eid0801.010176 11749746 PMC2730281

[21] Farrant JM, Drury AE, Thompson RP. Legionnaires' disease following immersion in river water. Lancet. 1988;2:460. 10.1016/S0140-6736(88)90456-4 2900394

[22] Nagai T, Sobajima H, Iwasa M, Tsuzuki T, Kura F, Amemura-Maekawa J, Neonatal sudden death due to *Legionella pneumophila* pneumonia associated with water birth in a domestic spa bath. J Clin Microbiol. 2003;41:2227–9. 10.1128/JCM.41.5.2227-2229.2003 12734286 PMC154682

[23] Franzin L, Scolfaro C, Cabodi D, Valera M, Tovo PA. *Legionella* pneumonia in a newborn after water birth: a new mode of transmission. Clin Infect Dis. 2001;33:e103–4. 10.1086/323023 11568855

[24] Institute of Environmental Science and Research Limited. Communicable Diseases Group Laboratory Services, Porirua (New Zealand). LabLink. 1998;5:31.

[25] Institute of Environmental Science and Research Limited. Communicable Diseases Group Laboratory Services, Porirua (New Zealand). LabLink. 1999;6:3.

[26] Institute of Environmental Science and Research Limited. Communicable Diseases Group Laboratory Services, Porirua (New Zealand). LabLink. 2000;8:4.

[27] Ratcliff RM, Lanser JA, Manning PA, Heuzenroeder MW. Sequence-based classification scheme for the genus *Legionella* targeting the *mip* gene. J Clin Microbiol. 1998;36:1560–7. 9620377 10.1128/jcm.36.6.1560-1567.1998 PMC104877

[28] Fry NK, Bangsborg JM, Bernarder S, Etienne J, Forsblorn B, Gaia V, Assessment of intercenter reproducibility and concordance of *Legionella pneumophila* serogroup 1 genotyping by amplified fragment length polymorphisms. Eur J Clin Microbiol Infect Dis. 2000;19:773–80. 10.1007/s100960000359 11117642

[29] Gaia V, Peduzzi R. Multi-locus sequence typing for characterization of *Legionella pneumophila* serogroup 1 isolates. In: Marre R, Abu Kwaik Y, Bartlett C, Cianciotto NP, Fields BS, Frosch M, et al., editors. *Legionella*. Washington: ASM Press; 2002. p. 251–3.

